# PaLS Study: How Has the COVID-19 Pandemic Influenced Physical Activity and Nutrition? Observations a Year after the Outbreak of the Pandemic

**DOI:** 10.3390/ijerph18189632

**Published:** 2021-09-13

**Authors:** Alicja Monika Jodczyk, Grzegorz Gruba, Zuzanna Sikora, Przemysław Seweryn Kasiak, Joanna Gębarowska, Natalia Adamczyk, Artur Mamcarz, Daniel Śliż

**Affiliations:** 1 Students’ Scientific Group of Lifestyle Medicine, 3rd Department of Internal Medicine and Cardiology, Medical University of Warsaw, 04-749 Warsaw, Poland; alicja.jodczyk@gmail.com (A.M.J.); gregorygpl@gmail.com (G.G.); zznnsikora@gmail.com (Z.S.); gebarowska.joanna@gmail.com (J.G.); adamczyk.natalia1997@gmail.com (N.A.); sliz.daniel@gmail.com (D.Ś.); 2 Polish Society of Lifestyle Medicine, 00-388 Warsaw, Poland; artur.mamcarz@wum.edu.pl; 3 3rd Department of Internal Medicine and Cardiology, Medical University of Warsaw, 04-749 Warsaw, Poland; 4 School of Public Health, Postgraduate Medical Education Center, 01-813 Warsaw, Poland

**Keywords:** physical activity, nutrition, public health, COVID-19 pandemic, lifestyle medicine

## Abstract

The restrictions implemented to prevent the spread of the SARS-CoV-2 virus have impacted the majority of life domains. To evaluate their potential consequences on physical activity (PA) and dietary habits among Polish undergraduates, a survey consisting of the IPAQ-SF, authors’ questions based on the Polish National Institute of Public Health recommendations, and authors’ questions scaled −5/0/5 on personal opinion was created and administered between 22 February and 3 April 2021. A total of 1323 students met the study conditions (1021 females, 297 males, 5 did not specify gender, mean age: 22 years old (SD = 4), mean BMI = 22.27 kg/m^2^ (SD = 3.87)). A total of 27.21% of students were in the low, 48.53% in the moderate, and 24.26% in the high PA group. A total of 71.94% estimated that the pandemic had a negative impact on their PA, 8.16% no impact, and 19.9% a positive impact. A total of 35.5% had an insufficient intake of vegetables, 34.3% declared adding salt to meals, and 31.6% ate animal-based products the majority of days in a week or every day. A total of 51.02% assessed the impact of the pandemic on their nutrition as negative, 20.11% did not notice changes, and 28.87% reported a positive impact on their dietary habits. Most of the students saw harmful pandemic effects on their diet and PA compared to the times before restrictions. This can lead to a higher prevalence of non-communicable diseases (NCDs) in the future.

## 1. Introduction

The outbreak of the COVID-19 pandemic has forced a change in the ordinary lifestyle of many populations. On 20 March 2020, the government announced the epidemic in Poland [1] and introduced numerous restrictions to limit the spread of the virus: staying at home, quarantine periods, and the closure of fitness clubs, gyms, and swimming pools. At the time, these actions were probably necessary [2,3], but they caused a significant decrease in exercise options and changed dietary habits [4]. Observations and the available literature indicate that these changes affected people’s lifestyle and could have an impact on medical problems we will be dealing with in the future [5,6]. Studies from different countries report that home confinement and other imposed safety measures have negatively influenced physical activity (PA) levels among many age groups [7,8,9], including university students [10,11]. In addition, previous research has reported negative changes in dietary patterns [12,13]. Studies show that chronic life stress seems to be linked with a greater intake of energy- and nutrient-dense foods, especially those with high sugar and fat [14], while also being associated with a higher BMI (Body Mass Index) [15].

The nutrition and level of physical activity of university students were alarming and unsatisfying before the pandemic [16,17]. In previous years, there has been a rise in the westernisation of diet among this population [18]. Moreover, the consumption of meat, animal and vegetable fat (margarine), and sugar [18] increased. Polish university students’ diet was characterised by a high intake of cured meat, smoked sausages, hot dogs, white bread, bakery products, butter, fried foods, and energy drinks [17]. What is more, students from Polish universities had the highest scores in the consumption of food of a potentially negative effect on health in comparison to students from Germany and Slovakia [17]. They often skipped breakfast, snacked in between main meals, and had insufficient levels of physical activity [19,20]. Data from previous studies show that the large majority of university students failed to meet recommendations on exercise and had inadequate PA levels [19,21,22]. Despite being aware of the importance of a proper diet and adequate levels of PA, students did not implement these rules into their lives [17,19].

Physical activity and a healthy diet have numerous benefits on health [23]. A higher adherence to the Mediterranean diet is associated with a lower incidence of type 2 diabetes, a lower incidence/mortality of CVD, and a lower incidence/mortality of cancer [24]. Being physically active improves all-cause mortality, cardiovascular disease mortality, incident hypertension, incident site-specific cancers, incident type 2 diabetes, mental health (reduced symptoms of anxiety and depression), cognitive health, and sleep [25]. Physical inactivity, obesity, and a Western diet are modifiable risk factors of most non-communicable diseases (NCDs) [26]. They increase mortality and cause a loss of disease-free years of life [27,28]. They are also associated with a higher risk of community-acquired pneumonia and pneumonia mortality [29]. A combination of a Western diet and physical inactivity results in an increased BMI [30], associated with a higher risk of diabetes, cardiovascular disease, joint disorders (osteoarthritis), and oncogenesis, especially endometrial, breast, ovarian, prostate, liver, gallbladder, kidney, and colon cancers [31]. NCDs are increasing worldwide and within the European region [32,33]. We believe that the COVID-19 pandemic and the lockdown may cause an increase in the number of people suffering from NCDs at a younger age.

Therefore, our study was a part of the PaLS—Pandemic against LifeStyle project, which aimed to evaluate the impact of the lockdown on the lifestyle of Polish university students. We hypothesised that the COVID-19 pandemic would have a negative impact on this population by decreasing their PA level, increasing sedentary behaviour, and causing poorer nutritional habits. To the best of our knowledge, no scientific data regarding the effect of the COVID-19 pandemic on the abovementioned lifestyle areas in the Polish undergraduate population have been reported.

## 2. Materials and Methods

### 2.1. Design and Selection of Study Subjects

**Figure 1:**
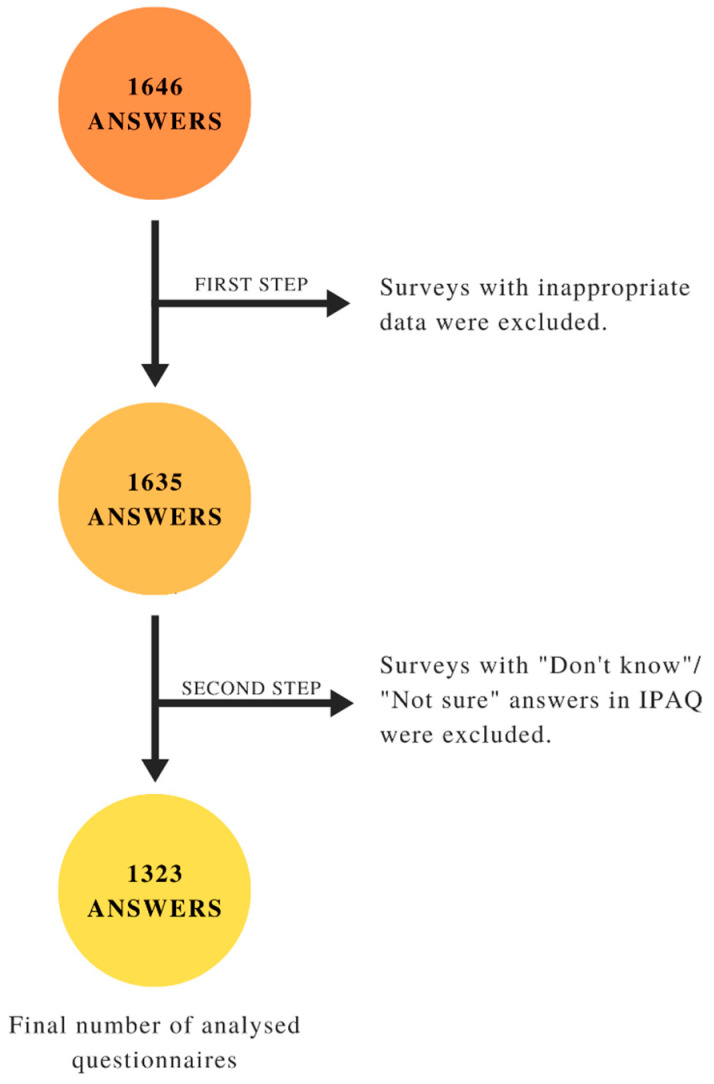
Stepwise data cleaning process.

A cross-sectional survey was designed and shared via different social media channels and e-mails (e.g., Polish Lifestyle Medicine newsletter, students’ university groups, Instagram, Facebook) a year after the outbreak of the pandemic in Poland from 22 February 2021 to 3 April 2021. It was half-term time and the beginning of the second academic semester. Answers were collected with the use of online survey app Google Sheets and converted into Excel file for analysis. Our study had only 2 inclusion criteria. Respondents had to: 1. be a 1st to 6th year of study university student (bachelor’s or master’s degree studies or equivalent), 2. study at a Polish university. There were no age limitations, but we did not include postgraduate and doctoral students. The requirements were pointed out at the beginning of the questionnaire so that the participants could assess if they fulfilled them or not. Data were collected from 1646 participants, and 1323 of them met all the study conditions. We excluded responders whose answers were unviable (e.g., BMI = 5 kg/m^2^ or being physically active for 24 h/day) or answered “I don’t remember” in any of the International Physical Activity—Short Form (IPAQ-SF) questions. There were no other exclusion criteria. Data were collected during the semester break and the beginning of the second semester. Figure 1 illustrates the number of excluded students at each part of our two-stage procedure.

### 2.2. Construction of the Questionnaire

A statement about the aims of the study and the declaration of anonymity and confidentiality were included in the form. Filling in the questionnaire and clicking the “send” button was tantamount to informed consent to participate in the study (proper information was mentioned at the background of the questionnaire). This study did not require Institutional Review Board approval and a proper judgement was obtained. Sociodemographic data such as age, sex, year of study, and self-reported biometric data height and weight were collected. Participants were asked about their physical activity and sedentary behaviour with questions from the International Physical Activity Questionnaire—Short Form (IPAQ-SF) [34] and eating habits with authors’ questions based on Polish National Institute of Public Health recommendations [35]. The survey contained an additional −5/0/+5 scaled questions about responders’ subjective assessment of the pandemic’s impact on their physical activity (PA) and eating habits. Questionnaire form is available in the Supplementary Material.

#### 2.2.1. IPAQ-SF and Dietary Habits

The IPAQ- SF was included to estimate the amount of time dedicated to PA per week. The questionnaire is adapted for many populations (including the Polish one). It assesses three specific types of activity: walking and moderate and vigorous intensity. Due to the scoring protocol, data are calculated into MET-minutes/week values [36]. MET (metabolic equivalent) is the specific metabolic equivalent and its values vary for each category of PA (3.3 MET for walking, 4 MET for moderate-intensity PA, and 8 MET for vigorous-intensity PA) [37]. To calculate weekly energy expenditure (MET-minutes/week values) for each type of activity, the following formula is used: weekly energy expenditure (MET-min/week) = MET x duration of PA type (minutes) x frequency. Total MET-minutes/week value is a sum of walking MET-min/week, moderate MET-min/week, and vigorous MET-min/week. According to scoring protocol, individuals are divided into PA categories: low, moderate, high. The final question in IPAQ-SF assesses the daily amount of time in a sitting position by obtaining it in hours and minutes.

To assess dietary changes, 12 authors’ questions based on the Polish National Institute of Public Health recommendations were designed [35]. Responders were asked about the composition of their meals and the supply of the nutrients (e.g., consumption of vegetables and fruits, unsweetened milk, products containing processed meat, products that are a source of animal fats or trans fatty acids, sweetened beverages or fruit juices instead of water, salt). Additionally, questions about dietary behaviours such as eating in front of electronic screens, paying attention to the nutritional value of buying products, and consuming more meals than before the pandemic were asked. For that part of the survey, participants could choose one of the following responses: once a week and less often, 2–3 times a week, most days of the week, every day. These figures were expressed as a percentage and compared with the Polish National Institute of Public Health recommendations.

#### 2.2.2. Self-Assessment Question

Participants matched the level of perceived change resulting from the pandemic in −5/0/+5 scaled questions. Answers from −5 to −1 meant that the pandemic had a negative impact, answer 0 meant no effect, and answers from +1 to +5 meant positive impact. It was an additional question in both parts: physical activity and eating habits.

### 2.3. Data Analysis

All statistical analyses were performed using the statistical software STATISTICA (version 13.3, StatSoft Polska Sp.z. o.o., Kraków, Poland) and SPSS Statistics (version 27.0, IBM, Chicago, IL, USA). Participants were classified into subgroups (age, sex, year of study, studying at medical or non-medical university). Based on mean answers and statistical test scores, the direction of correlation (positive or negative) was assessed. To calculate the correlation between studying at medical university/BMI, gender/PA level, BMI/PA level, studying at medical university/PA, and BMI/dietary habits, the U Mann–Whitney test was performed. Correlations between the year of study and reported level of perceived pandemic impact (declared answers in −5/0/+5 questions) were calculated based on the H Kruskal–Wallis test. For analysis of the correlation between studying at medical university/dietary habits, higher MET-min/week value, and reported pandemic impact in self-assessment (−5/0/+5) scale, rho-Spearman test and r-Pearson test were performed. Findings on eating habits were converted into percentage values and compared to Polish National Institute of Public Health recommendations.

## 3. Results

### 3.1. Sociodemographic Characteristics and BMI Index

A total number of 1646 Polish students participated in the study, and 1323 of them met the study conditions (1021 females, 297 males, 5 did not specify gender). The mean age of the participants was 22 years old (SD = 4), and the median was 22 years old. The demographic characteristics of participants including gender, BMI category, university, and year of study are presented in Table 1. Mean BMI was 22.27 kg/m^2^ (SD = 3.87); median 21.48 kg/m^2^ (SD = 3.87). The mean BMI of medical university students was 21.95 kg/m^2^ (SD = 3.37) and non-medical university students was 22.49 kg/m^2^ (SD = 4.26).

### 3.2. Results from International Physical Activity Questionnaire—Short Form

The results from the IPAQ-SF questionnaire are presented in Table 2.

Due to a scoring protocol, IPAQ-SF participants were divided into three categories of PA: high, moderate, and low, as presented in Table 3.

### 3.3. Dietary Habits Results

The frequency of the particular answers to diet-related questions is shown in Table 4.

### 3.4. Results of Self-Opinion Questions

#### 3.4.1. Results of Self-Opinion Question on Physical Activity

**Figure 2:**
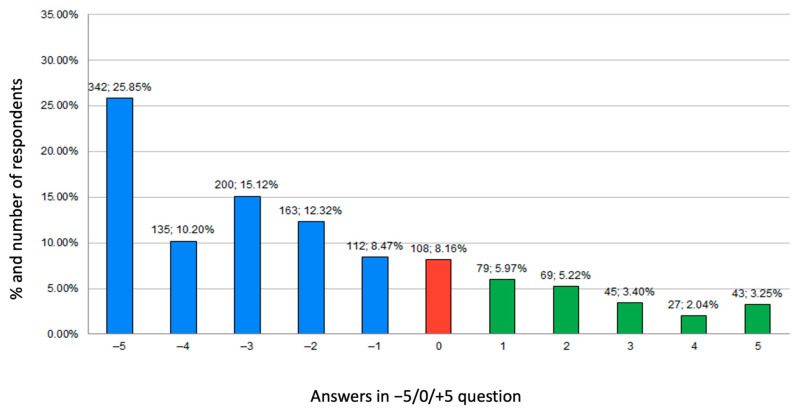
Answers to self-opinion questions on physical activity expressed in number of participants and percentages.

**Figure 3:**
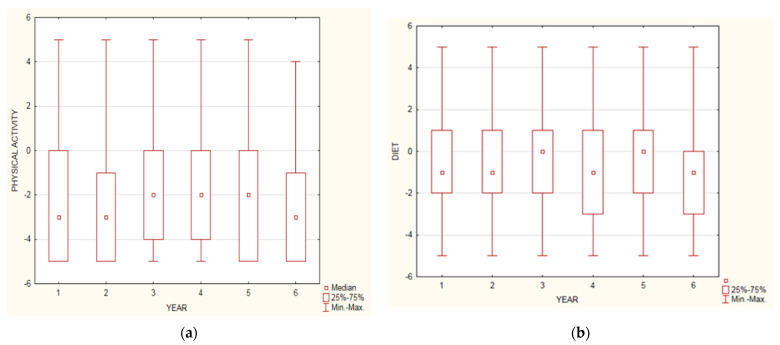
Results of self-opinion question depending on year of study on physical activity (**a**) and dietary habits (**b**): x—year of study, y—chosen answer (scale −5, 0, +5).

In summary, 71.94% of participants noticed a negative impact of the pandemic on their PA (answers from −5 to 0), 8.16% no effect (answer 0), and 19.9% a positive impact (answers from 0 to +5). A total of 25.85% of them estimated that the pandemic influenced their lifestyle in the worst possible way (answer −5). All data are presented in Figure 2. Independently of the year of study, students reported a comparably negative impact of the pandemic on their PA, as shown in Figure 3a (usage of Figure 3b is explained later in the Section 3.4.2).

#### 3.4.2. Results of Self-Opinion Question on Dietary Habits

**Figure 4:**
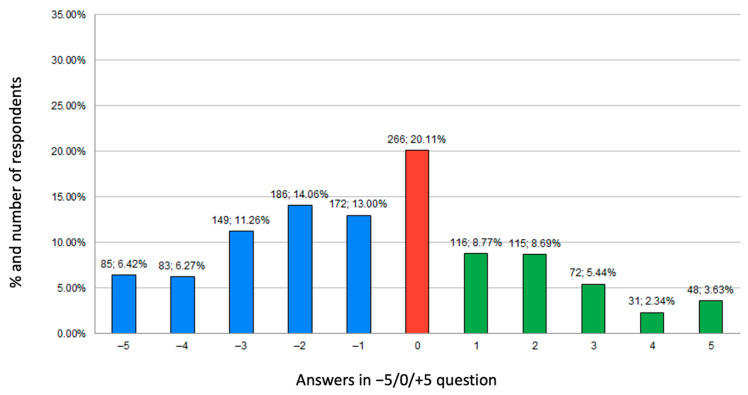
Answers to self-opinion question about dietary habits expressed in number of participants and percentages.

A total of 51.02% of the respondents assessed the impact of the COVID-19 pandemic on their dietary habits as negative (answers from −5 to 0), 20.11% considered that it has had no impact (answer 0), and 28.87% associated lockdown with a positive effect (answers from 0 to +5) on their nutritional behaviours. Answers on data on self-opinion questions are presented in Figure 4. Irrespective of the year of study, respondents declared a similar (moderately negative) impact of the COVID-19 pandemic on their dietary habits (data presented in Figure 3b).

### 3.5. Correlations

Statistical correlations obtained in the research are presented in Table 5. In the two right-end columns, additional information about the test score, odds ratio, and confidence interval is shown.

### 3.6. Summary of Differences in Results between Medical and Non-Medical Students

According to the results, medical university students paid more attention to their nutrition and physical activity. In comparison to non-medical university students, they statistically more often paid attention to labels of chosen products during shopping and took ingredients and the amount of calories into account. They consumed processed meat products and those that are a source of animal fats or trans fatty acids less often and replaced them with protein-rich plant products more often. They drank sweetened beverages or fruit juices instead of water and consumed less than 400 g of vegetables and fruits statistically less often. They also achieved higher values of MET-min/week values.

## 4. Discussion

Subjective assessment of the lifestyle habits of the participants revealed that the pandemic had a negative impact on their PA and nutrition. A total of 71.94% of them estimated the effect of the pandemic on their PA as negative (answers from −5 to −1). This was correlated with lower PA levels determined by IPAQ-SF. Students spent most of the day sitting. Previous studies show that the restrictions have reduced overall PA (number of days and number of hours) and access to exercise [9]. The decrease in the PA level might have been affected by different factors. The closure of gyms, fitness clubs, and swimming pools caused a significant decrease in exercise possibilities. It was previously reported that participation in sports clubs had an important position in PA promotion for younger populations and that sports club participants were more likely to meet PA recommendations [38]. Additionally, our study was conducted during winter and early spring, when the weather conditions were poor and possibilities of outdoor activities were limited. According to the results, students spent most of the day sitting, likely due to the increased time that people were required to stay within their quarantine location [9]. Universities transitioned to online learning and many places of students’ part-time jobs have been closed. These factors forced them to stay at home and could have caused an increase in sedentary behaviour.

There was a statistical correlation between lower MET-min/week values and a higher amount of sitting time. Lower PA is associated with a higher risk of all-cause mortality and hypertension, which is a key factor in cardiovascular disease [25]. There is a correlation between lower PA levels and a higher incidence of type 2 diabetes. Remaining physically active reduces symptoms of anxiety and depression and positively impacts cognitive health and sleep. Higher amounts of sedentary behaviour are connected with a higher risk of all-cause mortality, cardiovascular disease mortality, and the incidence of type 2 diabetes [25].

In our study, we investigated whether students’ dietary choices were compatible with “Healthy Nutrition Recommendations” developed by the National Institute of Hygiene [35]. We found that more than one-third of the study population (35.5%) had an insufficient intake of fruits and vegetables (less than 400 g per day) on most days of the week or every day. The importance of meeting this requirement and its function in preventing CVD diseases were observed in previous studies [39,40]. Only 12.7% of respondents reported that they consume sources of unsaturated fatty acids every day, while 22.1% of them declared eating products that are sources of animal fats on the majority of days within a week, and 9.5% every day. Guidelines emphasise that the consumption of animal sources of fat should be replaced by consuming vegetable oils. An improper balance of saturated and unsaturated fatty acids consumption poses a risk of the aggravation of dyslipidaemia and can increase the risk for a severe course of COVID-19 and disease mortality [41]. More than one-third of the responders (34.3%) declared adding salt to meals on the majority of days within a week or every day. A high salt content in the diet can result in developing hypertension. Many guidelines recommend reducing salt intake by avoiding adding additional salt to meals and reducing the consumption of processed foods such as meat products and salted snacks [42,43,44]. Negative changes in eating behaviours could be attributed to mood-driven eating (e.g., out of anxiety or boredom) and a dip in motivation to participate in PA or maintain healthy eating [9]. Acute and chronic stress is connected with impulsive, rewarding behaviour, such as overeating [15]. The high amount of students with an insufficient intake of fruits and a high intake of products that are sources of animal fats may be associated with the winter–spring season, when people generally consume food containing more calories [45]. It should be emphasised that even before the pandemic, university students’ dietary habits were far from being compliant with the nutritional recommendations for fruit, vegetables, and sodium intake [46].

An interesting finding from our study is that medical university students seemed to pay more attention to their dietary choices and achived higher MET-min/week values. This fact could be explained by the character of their universities and their academic environment. In general, medical university students should gain more knowledge about the benefits and necessity of healthy habits. These findings are not in accordance with the results observed in previous research, which suggested that the highest level of knowledge on food and nutrition was not connected with a healthier diet [16,17]. Other studies revealed that even students from medical and nutritional university departments showed inadequate awareness on healthy eating habits [16] and did not fulfil the PA and nutritional recommendations [19].

We found some correlations between PA and BMI as well as between nutrition and BMI. Participants with a higher BMI had a lower PA level, consumed more meals per day than before the pandemic, consumed products that are a source of animal fats or trans fatty acids more often, and replaced water with sweetened beverages or fruit juices more often. The correlation between a higher BMI and poorer dietary choices was also found in a different study among adults in Poland [47]. Obesity is often considered to be a result of either excessive food intake or of insufficient physical activity [48]. Matching energy intake to a level of energy expenditure is an important strategy to maintain a healthy weight [48]. There is a great need to evaluate the potential impact of teaching young populations about energy balance (i.e., how energy in food interacts with energy expenditure to determine body weight) and about how food and physical activity choices impact energy balance [48].

Pre-pandemic studies show that university students did not fulfil the recommendations for PA and nutrition before [20]. This trend might continue and intensify. University is the last stage in young people’s lives, in which they have a chance to acquire proper knowledge and healthy habits before beginning an independent adult life [49,50]. Therefore, proper actions should be taken to encourage young people to live a healthy lifestyle [51].

## 5. Conclusions

A high number of Polish students did not meet nutritional recommendations. Moreover, many of them did not meet physical activity requirements described as for medium or highly active people. Most of them declared a negative impact on their PA and diet due to the COVID-19 pandemic. Effects of physical inactivity and bad dietary habits caused by the pandemic may have long-term consequences and can lead to the deterioration of health and chronic diseases.

For a better assessment of the impact of the COVID-19 pandemic on the physical activity of Polish students, more studies are needed. An in-depth analysis of these issues is necessary to evaluate and implement interventions.

## 6. Limitations

The presented study was conducted online; therefore, we cannot be sure that the responders fully understood the questions and answered them correctly. The outcomes could be affected by the fact that our form was more likely fulfilled by people interested in a healthy lifestyle. The majority of our responders were females. This could be related to the high number of females in universities in Poland [52]. In addition, women in Poland were more likely to discuss the topic of the COVID-19 pandemic on Facebook and Instagram, which were the platforms where our survey was mainly distributed [53]. The study was conducted in a relatively short period of time, during winter and early spring, which could have influenced the results of IPAQ-SF—during winter, people normally tend to participate in less sport. There is a probability that physical activity and dietary habits varied during the pandemic. In addition, data from times before restrictions were imposed were not collected. This was due to the fact that our study was conducted nearly a year after the outbreak of the pandemic and there was a high probability that the responders would not have remembered their habits after this time. To decrease the impact of the limitations, we implicated the data clinic process with the exclusion of unviable answers with a high probability of misunderstanding the questions. A limitation that must be highlighted is the fact that it was not possible to demonstrate the validity of the questionnaire through common analysis methods (i.e., using objective measures of PA from movement devices such as accelerometers or pedometers for comparison with the self-reported measures).

## Figures and Tables

**Table 1:** Demographic characteristics of participants.

Variables		Number	%
Sample	Participants Female Male Did not specify gender	1323 1021 297 5	100 77.17 22.45 0.38
BMI category	Underweight (BMI < 18.5 kg/m^2^) Normal weight (BMI 18.5–25 kg/m^2^) Overweight (BMI 25–30 kg/m^2^) Obese (BMI ≤ 30 kg/m^2^)	154 927 188 54	11.64 70.07 14.21 4.08
University	MUS NMUS	630 693	47.62 52.38
Year of study	1st 2nd 3rd 4th 5th 6th	442 241 207 207 177 49	33.4 18.22 15.65 15.65 13.38 3.7

Abbreviations: MUS—medical university students, NMUS—non-medical university students.

**Table 2:** IPAQ-SF results.

Type of Activity		Females	Males	Total
VPA	Days/week Min/week MET/week	1.81 ± 1.75 71.06 ± 101.59 565.19 ± 812.7	2.06 ± 1.88 112.48 ± 161.63 899.85 ± 1293.07	1.87 ± 1.78 80.49 ± 119.11 640.6 ± 952.86
MPA	Days/week Min/week MET/week	2.14 ± 1.81 72.53 ± 93.44 290.12 ± 373.77	2.31 ± 2.01 106.99 ± 162.67 427.97 ± 650.7	2.18 ± 1.86 80.3 ± 113.73 321.18 ± 454.92
Walking	Days/week Min/week MET/week	4.07 ± 2.15 157.79 ± 174.39 520.72 ± 575.46	4.08 ± 2.17 180.16 ± 215.02 594.52 ± 709.58	4.07 ± x 162.83 ± 184.56 537.35 ± 609.05
All PA	Days/week Min/week MET/week	8.02 ± 4.33 300.97 ± 276.03 1376.03 ± 1376.03	8.44 ± 4.81 399.63 ± 447.47 1922.35 ± 2221.36	8.11 ± 4.44 323.20 ± 325.33 1499.14 ± 1579.21
Sitting time	Min/week	559.2 ± 196.2	561 ± 191.4	559.2 ± 194.4

Abbreviations: VPA—vigorous physical activity, MPA—moderate physical activity.

**Table 3:** Physical activity categories based on IPAQ-SF scoring protocol; MUS—medical university students, NMUS—non-medical university students.

Group of Students	Low	Moderate	High
Males: MUS	18 (17.65%)	54 (52.94%)	30 (29.41%)
Males: NMUS	58 (29.74%)	78 (40.00%)	59 (30.26%)
Females: MUS	161 (32.59%)	226 (45.75%)	107 (21.66%)
Females: NMUS	123 (23.34%)	280 (53.13%)	124 (23.53%)
Did not specify gender	0 (0%)	4 (80%)	1 (20%)
Total	360 (27.21%)	642 (48.53%)	324 (24.26%)

Abbreviations: MUS—medical university students, NMUS—non-medical university students.

**Table 4:** Incidence within a week (% of responders) of particular dietary habits during COVID-19 pandemic.

	Once a Week or Less Often (%)	2–3 Times Per Week (%)	Majority of Days within a Week (%)	Everyday (%)
Consuming more meals per day than before the pandemic (including snacking):	42.86	26.98	22.68	7.48
Consuming less than 3 servings of wholegrain products daily (less than 90 g/day):	40.8	27.7	22.4	9.2
Consuming less than 400 g vegetables and fruits:	34.0	30.5	22.7	12.8
Consuming less than 2 glasses of unsweetened milk or other dairy products daily:	38.9	24.8	19.7	16.7
Consuming products containing processed meat, such as sausages, ham, frankfurters, etc.:	43.5	27.7	19.1	9.8
Replacing meat with protein-rich plant products such as nuts and legumes: beans, chickpeas, soy, lentils, fava beans, peas:	51.9	21.5	15.4	11.2
Consuming products that are sources of animal fats or trans fatty acids present in products, such as pastries, candy bars, salty snacks, and fast-food products:	31.6	36.9	22.1	9.5
Consuming products that are sources of unsaturated fatty acids, such as canola oil, olive oil, or fish:	20.1	38.0	29.2	12.7
Drinking sweetened beverages or fruit juices instead of water:	20.1	38.0	29.2	12.7
Adding salt to meals:	41.5	24.3	20.0	14.3
Consuming meals while looking at the screen of a TV, computer, or other device:	9.6	17.0	34.2	39.2
Paying attention to labels of chosen products during shopping, taking into account ingredients, amount of calories, etc.	36.5	20.4	23.0	20.1

**Table 5:** Correlations.

Type of Variable	Higher BMI	Studying at MU	Lower Score in PA Self-Opinion Question
Lower level of PA	*p* = 0.017	-	*p* < 0.001; r = 0.4; Rho = 0.435
Higher level of PA	-	*p* = 0.014	-
Consumption of more meals before pandemic per day	*p* < 0.001	-	-
Higher consumption of animal fats or trans fatty acids	*p* = 0.003	-	-
More frequent replacement of water with sweetened beverages or fruit juices	*p* = 0.014	-	-
More frequent attention paid to labels of products and their caloric intake	-	*p* < 0.001; OR = 1.784; 95%Cl = 1.432–2.222	-
Less frequent consumption of processed meat products	-	*p* < 0.001; OR = 0.544; 95%Cl = 0.426–0.695	-
Less frequent consumption of animal fats and trans fatty acid sources	-	*p* = 0.007; OR = 0.727; 95%Cl = 0.575–0.919	-
More frequent replacements of animal protein sources with plant-based equivalents	-	*p* < 0.001; OR = 1.736; 95%Cl = 1.357–2.221	-
Less frequent drinking of sweetened beverages or fruit juices instead of water	-	*p* < 0.001; OR = 0.331; 95%Cl = 0.24–0.455	-
Less frequent consumption of less than 400 g vegetables and fruits per day	-	*p* = 0.019; OR = 0.763; 95%Cl = 0.608–0.957	-

Abbreviations: MU—medical university; PA—physical activity.

## Data Availability

The data presented in this study are available on request from the corresponding author. The data are not publicly available due to not obtaining consent from respondents for publishing the data.

## Supplementary Materials

The following are available online at https://www.mdpi.com/article/10.3390/ijerph18189632/s1, S1: Questionnaire form.
