# Supplementary material for: PaLS Study: How Has the COVID-19 Pandemic Influenced Physical Activity and Nutrition? Observations a Year after the Outbreak of the Pandemic
Source: Int J Environ Res Public Health. 2021 Sep 13;18(18):9632. doi: 10.3390/ijerph18189632 (PMC8470829; doi:10.3390/ijerph18189632)
Supplement: Supplementary file 1 [file ijerph-18-09632-s001.zip › ijerph-1349596-supplementary.pdf]

Supplementary material- Questionnaire form

ENGLISH Version

Part 1.- general and demographic questions

1. Gender

_____(male, female, other, I would rather not to give it)

2. Height

_____(in cm)

3. Weight

_____(in kg)

4. Age

_____(in years)

5. Are you a Medical University student? (fields which include you into Medical University students: medicine, stomatology, pharmacy, physiotherapy, nursing, paramedics, dietetics etc.)?

_____(yes/no)

6. Year of studies

_____(1-6)

Part 2.- physical activity questions (IPAQ-SF)

Think about all the vigorous activities that you did in the normal pandemic week. Vigorous physical activities refer to activities that take hard physical effort and make you breathe much harder than normal. Think only about those physical activities that you did for at least 10 minutes at a time.

Q1. During the normal pandemic week, on how many days did you do vigorous physical activities like heavy lifting, digging, aerobics, or fast bicycling?

_____ days per week

If no vigorous physical activities skip to question 3.

Q2. How much time did you usually spend doing vigorous physical activities on one of those days?

_____ hours per day
_____ minutes per day

- Don’t know/Not sure

Think about all the moderate activities that you did in the normal pandemic week. Moderate activities refer to activities that take moderate physical effort and make you breathe somewhat harder than normal (like carrying light loads, bicycling at a regular pace, or doubles tennis). Do not include walking. Think only about those physical activities that you did for at least 10 minutes at a time.

Q3. During the normal pandemic week, on how many days did you do moderate physical activities?

_____ days per week

If no moderate physical activities Skip to question 5.

Q4. How much time did you usually spend doing moderate physical activities on one of those days?

_____ hours per day
_____ minutes per day

- Don’t know/Not sure

Think about the time you spent walking in the normal pandemic week. This includes at work and at home, walking to travel from place to place, and any other walking that you have done solely for recreation, sport, exercise, or leisure.

Q5. During the normal pandemic week, on how many days did you walk for at least 10 minutes at a time?

_____ days per week

If no walking Skip to question 7.

Q6. How much time did you usually spend walking on one of those days?

_____ hours per day
_____ minutes per day

- Don’t know/Not sure

The last question is about the time you spent sitting on weekdays during the normal pandemic week. Include time spent at work, at home, while doing course work and during leisure time. This may include time spent sitting at a desk, visiting friends, reading, or sitting or lying down to watch television.

Q7.During the normal pandemic week, how much time did you spend sitting on a week day?

_____ hours per day
_____ minutes per day

- Don’t know/Not sure

Part 3.- diet questions

	Once a week or less often (%)	2-3 times per week (%)	Majority of days within a week (%)	Everyday (%)
Consuming more meals per day than before the pandemic (including snacking)?				
Consuming less than 3 servings of wholegrain products daily (less than 90g/day)?				
Consuming less than 400g vegetables and fruits?				
Consuming less than 2 glasses of unsweetened milk or other dairy products daily?				
Consuming products containing processed meat, such as sausages, ham, frankfurters etc.?				
Replacing meat by protein rich plant products such as nuts and legumes: beans, chickpeas, soy, lentils, fava bean, peas?				

**Consuming products which are source of animal fats or trans fatty acids present in products, such as pastries, candy bars, salty snacks and fast-food products?**	
**Consuming products, which are source of unsaturated fatty acids, such as canola oil, olive oil or fish?**	
**Drinking sweetened beverages or fruit juices instead of water?**	
**Using additional salt to meals?**	
**Consuming meals while looking at the screen of TV, computer or other devices?**	
**Paying attention to labels of chosen products during shopping, taking into account ingredients, amount of calories etc.?**	

POLISH Version

Część 1.- pytania ogólne i demograficzne

1. Płeć

_____(mężczyzna, kobieta, inna, wolę nie podawać)

2. Wzrost

_____(w cm)

3. Waga

_____(kg)

4. Wiek

_____(lata)

5. Czy jesteś studentką/studentem kierunku medycznego? (kierunek lekarski, lekarsko-dentystyczny, farmacja, fizjoterapia, pielęgniarstwo, ratownictwo medyczne, dietetyka itp.)?

_____ (tak/nie)

6. Rok studiów

_____ (1-6)

Część 2.- pytania dotyczące aktywności fizycznej (IPAQ-SF)

Na początek proszę przypomnieć sobie wszystkie czynności wymagające intensywnego wysiłku fizycznego.
Intensywny wysiłek fizyczny wywołuje bardzo szybkie oddychanie i bardzo szybkie bicie serca. Intensywnego wysiłku fizycznego wymaga np. dźwiganie ciężkich przedmiotów, kopanie ziemi, aerobik, szybki bieg, szybka jazda rowerem. Interesują nas tylko czynności, które trwały co najmniej 10 min. bez przerwy.

Q1. Przez ile dni w ciągu zwykłego tygodnia w trakcie pandemii wykonywałaś/eś takie czynności?

_____ dni w tygodniu

Jeżeli nie wykonywałaś/eś w ogóle intensywnego wysiłku fizycznego przejdź do pytania 3.

Q2. Przeciętnie ile czasu wykonywałaś/eś w ciągu jednego z tych dni czynności wymagające intensywnego wysiłku fizycznego?

_____ godzin w ciągu dnia
_____ minut w ciągu dnia

- Nie jestem w stanie określić

A teraz proszę przypomnieć sobie wszystkie czynności wymagające umiarkowanego (średniego) wysiłku fizycznego.
Umiarkowany wysiłek fizyczny prowadzi do trochę szybszego oddychania i trochę szybszego bicia serca
Umiarkowanego wysiłku fizycznego wymaga np. noszenie lżejszych ciężarów, jazda rowerem w normalnym tempie, gra w siatkówkę lub bardzo szybki marsz. Proszę

jednak nie brać pod uwagę chodzenia. Mamy na myśli ponownie tylko czynności, które trwały co najmniej 10 minut bez przerwy.

Q3. Zaznacz, w trakcie ilu dni w ciągu zwykłego tygodnia w trakcie pandemii uprawiałaś/eś umiarkowany wysiłek fizyczny?

_____ dni w tygodniu

Jeżeli nie wykonywałaś/eś w ogóle umiarkowanego wysiłku fizycznego przejdź do pytania 5.

Q4. Przeciętnie ile czasu wykonywałaś/eś w ciągu jednego z tych dni czynności wymagające umiarkowanego wysiłku fizycznego?

_____ godzin w ciągu dnia
_____ minut w ciągu dnia

- o Nie jestem w stanie określić

Teraz proszę przypomnieć sobie, ile czasu zajęło Panu/Pani chodzenie w ciągu jednego dnia w zwykłym tygodniu w trakcie pandemii. Interesuje nas chodzenie związane z pracą, chodzenie ulicą, np. po zakupy, do pracy, a także o spacery.

Q5. W trakcie ilu dni w ciągu zwykłego tygodnia w trakcie pandemii chodziłaś/eś co najmniej 10 min bez przerwy?

_____ dni w tygodniu

Jeżeli nie chodziłaś/eś przed co najmniej 10 minut przejdź do pytania 7.

Q6. Przeciętnie ile czasu chodziłaś/eś w ciągu jednego z tych dni co najmniej 10 min bez przerwy?

_____ godzin w ciągu dnia
_____ minut w ciągu dnia

- o Nie jestem w stanie określić

Ostatnie pytanie będzie dotyczyło czasu spędzanego w pozycji siedzącej. Ile czasu w ciągu zwykłego tygodnia w trakcie pandemii spędzał Pan/Pani siedząc? Tym razem proszę uwzględnić tylko dni powszednie, tzn. proszę pominąć sobotę i niedzielę. Chodzi np. o siedzenie przy biurku, siedzenie podczas odwiedzin u znajomych, podczas czytania, a także siedzenie lub leżenie podczas oglądania telewizji. Proszę uwzględnić czas spędzony na siedzeniu w domu, w pracy, w szkole, w pojazdach i w innych miejscach.

Q7. Ile czasu w ciągu dnia (średnio) w ciągu zwykłego tygodnia w trakcie pandemii spędziłaś/eś siedząc?

_____ godzin w ciągu dnia

_____ minut w ciągu dnia

- Nie jestem w stanie określić

Część 3.- pytania dotyczące diety

	Raz w tygodniu i rzadziej (%)	2-3 razy w tygodniu(%)	Przez większość dni w tygodniu (%)	Codziennie (%)
**Spożywanie większej liczby posiłków w ciągu dnia niż przed pandemią? (przez posiłki należy rozumieć również przekąski)**				
**Dziennie spożywałaś/eś mniej niż trzy porcje produktów pełnoziarnistych? (mniej niż 90g/dzień)**				
**Dziennie spożywałaś/eś mniej niż 400g warzyw i owoców?**				
**Dziennie spożywałaś/eś mniej niż 2 szklanki**				

niesłodzonego mleka lub innych przetworów mlecznych? (jednej szklance mleka odpowiada szklanka jogurtu, kefiru, maślanki lub ok. 6 grubych plastrów sera białego)	
Zjadałaś/eś produkty z przetworzonego mięsa? (przez mięso przetworzone należy rozumieć parówki, kiełbasy, szynki itd.)	
Mięso zastępowałaś/eś białkowymi produktami pochodzenia roślinnego? (tj. nasionami roślin strączkowych: fasola, ciecierzyca, soja, groch, soczewica, bób i orzechami)	
Źródłem tłuszczu w Twojej diecie były tłuszcze pochodzenia zwierzęcego lub częściowo utwardzone tłuszcze roślinne	

obecne m.in. w ciastkach, batonikach, słonych przekąskach i żywności typu fast-food?

Źródłem tłuszczu w Twojej diecie były tłuszcze roślinne (tj.olej rzepakowy, oliwa z oliwek) lub ryby?

Zamiast wody sięgałaś/eś po słodkie napoje lub soki owocowe?

Dosalałaś/eś posiłki?

Jadłaś/eś wpatrując się w ekran telewizora, komputera lub innych urządzeń?

Zwracałaś/eś uwagę (pod względem składu, ilości kalorii itp.) na etykiety kupowanych produktów?
